# Effects of Dietary Glutamine Supplementation on Heat-Induced Oxidative Stress in Broiler Chickens: A Systematic Review and Meta-Analysis

**DOI:** 10.3390/antiox12030570

**Published:** 2023-02-24

**Authors:** Chris Major Ncho, Vaishali Gupta, Yang-Ho Choi

**Affiliations:** 1 Department of Animal Science, Gyeongsang National University, Jinju 52828, Republic of Korea; 2 Institute of Agriculture and Life Sciences, Gyeongsang National University, Jinju 52828, Republic of Korea; 3 Division of Applied Life Sciences (BK21 Plus Program), Gyeongsang National University, Jinju 52828, Republic of Korea

**Keywords:** glutamine, heat stress, oxidative stress, broilers, meta-analysis, review

## Abstract

In avian species, heat stress (HS) is usually the result of being exposed to high ambient temperatures, whereas oxidative stress (OS) results from the overproduction of reactive oxygen species. The current literature suggests that HS often leads to OS. Therefore, this systematic review and meta-analysis was conducted to assess the effects of dietary supplementation of glutamine on the antioxidant status and growth performances in heat-stressed broilers. A total of 13 studies were deemed eligible after an exhaustive search of the literature from Google Scholar, PubMed, and Scopus. Briefly, the following criteria were used to select the studies: trials performed on broilers; publication in peer-review journals using English as the text language; and sufficient details about the design and inclusion of dietary glutamine as a treatment for HS. Two main categories of outcomes were extracted from the studies included in the review: growth parameters and OS markers. For the meta-analysis, a random effect model was used when the heterogeneity was higher than 50%, and a fixed effect model was applied otherwise. Pooled standardized mean differences (SMD), and mean differences (MD) with their confidence intervals (CI) from the studies revealed that dietary glutamine could increase body weight gain (SMD = 0.70, CI = 0.50 to 0.90, *p* < 0.05), and feed intake (FI) (SMD = 0.64, CI = 0.43 to 0.86, *p* < 0.05), and reduce the feed conversion ratio (MD = −0.05, CI = −0.07 to −0.02, *p* < 0.05) in heat-exposed birds. Additionally, higher glutamine (SMD = 1.21, CI = 1.00 to 1.43, *p* < 0.05), glutathione (SMD = 1.25, CI = 0.88 to 1.62, *p* < 0.05), superoxide dismutase (SOD) (SMD = 0.97, CI = 0.58 to 1.36, *p* < 0.05), and catalase (SMD = 0.94, CI = 0.72 to 1.16, *p* < 0.05) levels were recorded in the serum, breast, and thigh muscle after supplementation of glutamine. Furthermore, the subgroup analysis revealed that malondialdehydes levels were decreased only in the serum (SMD = −0.83, CI = −1.25 to −0.41, *p* < 0.001) and thigh muscle (SMD = −1.30, CI = −1.86 to −0.35, *p* < 0.001) while glutathione peroxidase (GPX) activity was increased in the breast (SMD = 1.32, CI = 0.95 to 1.68, *p* < 0.05) and thigh muscle (SMD = 1.53, CI = 1.06 to 1.99, *p* < 0.05). Meta-regression models indicated that longer periods of heat exposure were inversely associated with the effectiveness of dietary glutamine in increasing FI, GPX, and SOD (*p* < 0.05). Besides, increasing the dietary concentration of glutamine led to higher GPX and SOD levels (*p* < 0.05). Taken together, results suggest that dietary supplementation of glutamine can effectively mitigate the deleterious effects of HS by enhancing the antioxidant status and increasing growth performances in broilers.

## 1. Introduction

Over the years, intensive genetic selection has improved the productivity of commercial poultry. Indeed, the recent literature has reported around twenty broiler strains commonly used in the industry [1]. However, fast-growing strains such as Ross, Cobb, Hubbard, and Arbor Acres are the most popular worldwide. The high level of performance resulting from genetic selection has also affected bird health and immunity [2]. Current commercial broilers and layers are more prone to abiotic stress than those of the past. Climatic changes, along with other stressors in the environment, have led to the birds being reared in challenging conditions. Further, the ban on sub-therapeutic feeding of antibiotics in poultry almost globally and an ever-increasing demand for higher productivity of the birds in a fast competitive market have made commercial poultry farming more challenging than ever before [3]. Feed additives and supplements have been explored to elicit production and ameliorate stress in birds.

**Figure 1:**
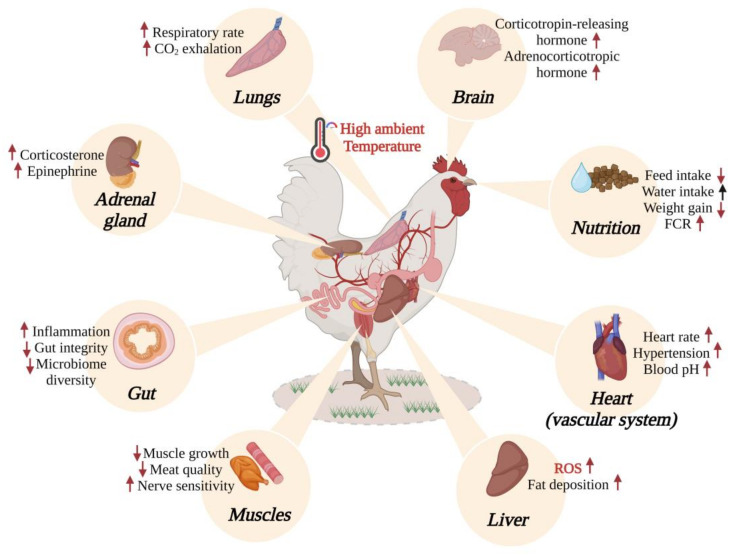
Selected established or suggested effects of high ambient temperatures in broilers. The figure has been realized based on information obtained from previous reports [5,19,20]. Upwards arrows indicate an increase and downward arrows indicate a decrease. Abbreviation: ROS: reactive oxygen species.

Heat stress (HS) occurs when the net heat flow from an organism to its surroundings is not equal to the heat generated within that organism’s body [4]. During HS, the hypothalamic–pituitary axis (HPA) is stimulated, causing a cascade of hormonal release [5]. More precisely, the hypothalamus releases the corticotrophin-releasing hormone (CRH), which in turn provides an impetus for the secretion of adrenocorticotropic hormone (ACTH) from the anterior pituitary, ultimately leading to the release of corticosteroids from the adrenal cortex [6]. Corticosterone further causes glycogenesis and gluconeogenesis [7]. Besides, HS is also associated with harmful effects on the respiratory system. Indeed, as the birds pant to release extra heat, excessive CO_2_ is lost during the process, leading to a disturbed acid–base balance [8]. In addition to being cytotoxic, HS adversely affects growth performance, intestinal development, and immunity by influencing digestive enzyme activity, and absorption of nutrients [9,10,11,12]. In fact, birds under HS spend more time drinking whereas feeding decreases, thereby reducing feed efficiency [13]. HS also affects the absorption, utilization, and metabolism of nutrients in the gut, and increases enteric diseases in poultry [14,15]. Another drawback of HS resides in its oxidative stress-inducing effects. Indeed, HS stimulates the production of a large number of reactive oxygen species and free radicals in skeletal muscles, which affects meat quality in broilers [16,17]. During acute HS, enterocyte proliferation and activity of the brush-border membrane enzymes in young birds are impaired [18], resulting in decreased digestibility of various essential amino acids and glutamine. Hence, high environmental temperatures lead to glutamine deficiency, causing a decrease in feed consumption, live weight, and feed efficiency in broilers. An overview of the overall harmful effects of HS in broilers has been summarized in Figure 1.

Glutamine is a conditionally essential amino acid that is synthesized in the liver and skeletal muscles in optimal body conditions. Moreover, it acts as an important precursor of glutamate and glutathione, which further is a component of the antioxidant enzyme glutathione peroxidase [16]. HS decreases the synthesis of glutamine and, to provide the body with an optimal level, exogenous glutamine can be supplemented in the diet to combat the deleterious effects of glutamine deficiency. In addition, dietary glutamine effectively reduces malondialdehydes (MDA) levels in the breast and thigh muscles of broilers during HS [21,22]. Supplementation of glutamine has also been shown to increase glutathione peroxidase (GPX) and catalase (CAT) and raised their gene expression levels in the breast muscle of broilers during HS [23].

Although extensive trials have been conducted on glutamine supplementation in heat-stressed broilers, studies on the subject have explored different setups and treatment concentrations. Therefore, it is important to summarize the previously published literature to evaluate the HS-alleviating effect of glutamine. The current meta-analysis was conducted to assess the potential effects of dietary glutamine supplementation on heat-induced oxidative stress in broilers. Effect sizes of growth and antioxidant parameters were pooled using random or fixed-effects models. Meta-regressions were also performed to evaluate associations between HS duration, dietary glutamine concentration, and response parameters included in this study.

## 2. Materials and Methods

This meta-analysis has been conducted following the CAMARADES (Collaborative Approach to Meta-Analysis and Review of Animal Data from Experimental Studies) guidelines for systematic review and meta-analysis [24]. The systematic review protocol was registered on the Open Science Framework platform on 2 January 2023 and is accessible via the following DOI: https://doi.org/10.17605/OSF.IO/TR7BJ.

### 2.1. Search Strategy

The relevant studies included in the meta-analysis were collected in June 2021. The research was carried out by consulting Google Scholar, PubMed, and Scopus. The corresponding queries were used to retrieve studies from the following databases and registers:

PubMed: “(glutami*) AND (heat OR high OR hot OR tropical) AND (temperature OR stress OR condition OR weather) AND (chick* OR poultry OR broiler* OR bird*)”.

Scopus: “TITLE-ABS-KEY ((glutami*) AND (heat OR high OR hot OR tropical) AND (temperature OR stress OR condition OR weather) AND (chick* OR poultry OR broiler* OR bird*))”.

In Google Scholar, the advanced search function was used to specify keywords to be found in the title of the articles. The corresponding query was: “allintitle: glutamine broiler”.

Two independent researchers performed the study research and collection. No authors were contacted to ascertain further information or to obtain unpublished data. The studies were selected after analyzing the title, abstract, and finally the full text. Studies were included in the database only if both researchers agreed to do so.

### 2.2. Selection Criteria

The studies were included in the meta-analysis only if the following criteria were satisfied: (i) studies were published in a peer-review journal; (ii) English was the language of the main text; (iii) broilers were used as experimental animals; (iv) the study design included a control treatment under HS conditions; (v) all HS parameters (HS category, temperature, duration, period) were available in the material and methods (vi); the treatments include at least dietary supplementation of glutamine only (studies that only evaluated the combination of glutamine and other nutrients were not eligible); and (vii) the concentration of dietary glutamine used was consistent all over the trial. Finally, articles that provided a duplicate set of data previously reported in another study were not included.

### 2.3. Study Quality Assessment

The potential risk of bias in the studies included in the meta-analysis was evaluated via the SYRCLE risk of bias tool for animal studies [25]. The study quality assessment was performed by two independent researchers [26]. The assessment points were related to sequence generation, baseline characteristics, allocation concealment, random housing, blinding, random outcome assessment, incomplete outcome data, selective outcome reporting, and other sources of bias.

### 2.4. Data Extraction and Processing

Two main categories of response variables were extracted from studies. The first category was the growth parameters, which included: body weight gain (BWG), feed intake (FI), and feed conversion ratio (FCR). The second category named antioxidant parameters included glutathione peroxidase (GPX), superoxide dismutase (SOD), catalase (CAT), malondialdehydes (MDA), and glutathione (GSH). Finally, glutamine (GLN) was also considered as an outcome. Mean, standard deviations (calculated based on standard error when not available), and sample size data from the treatments and control heat-stressed groups were extracted. When results were reported in graphs, WebPlotDigitizer (version 4.4) was used to retrieve the data. For studies including more than one treatment, each treatment was considered a separate trial. Therefore, in the forest plots, the name of a study could be followed by a letter (a,b…) depending on the number of treatments included. In addition, when two separate studies were published by the same author within the same year and included more than one treatment, a letter and a number (a1, a2, b1, b2…) were assigned to facilitate understanding by the reader. Data were then entered in an Excel file, with each row containing the name of the study as well as the mean, the sample size, the standard deviation of the treatment, and the control heat-stressed group for each parameter analyzed in the study.

### 2.5. Statistical Analysis

The realization of figures and the statistical analysis were executed using the package “metafor” of the R software version 4.1.0 (R Core Team, 2021). Due to the distinct nature of the variables, BWG, FI, GPX, SOD, CAT, GSH, MDA, and GLN were computed using the standardized mean difference (SMD) [27]. FCR was computed using the mean difference (MD) [28]. Indeed, SMD has been the preferred effect size for reporting continuous outcomes measured in different units. MD on the other side is appropriate for outcomes reported on the same scale across studies. For each variable x analyzed in the study, the following formulas were used for the calculation of the effect sizes [29]:

(1) $${\mathrm{MD}}_{x}={\bar{x}}_{1}-{\bar{x}}_{2}\;$$

(2) $${\mathrm{SMD}}_{x}=\frac{{\bar{x}}_{1}-{\bar{x}}_{2}}{s_{\mathrm{pooled}}}\;$$

with:

(3) $$s_{\mathrm{pooled}}=\sqrt{\frac{\left(n_{1}-1\right)s_{1}^{2}+\left(n_{2}-1\right)s_{2}^{2}}{\left(n_{1}-1\right)+\left(n_{2}-1\right)}}\;$$

where:

${\bar{x}}_{1}$ and ${\bar{x}}_{2}$ are the arithmetic means of the heat-stressed glutamine-treated and the heat-stressed control groups, respectively;

$n_{1}$ and $n_{2}$ are the sample size of the heat-stressed glutamine-treated and the heat-stressed control groups, respectively; and

$s_{1}$ and $s_{2}$ are the standard deviations of the heat-stressed glutamine-treated and the heat-stressed control groups, respectively.

In a meta-analysis, the extent of variation of the true effect sizes between studies, called heterogeneity, is an important parameter to take into consideration. Heterogeneity between trials was evaluated by the calculation of Cochran’s *Q* and the I^2^ statistic. Traditionally, Cochran’s *Q* is used to differentiate “real” between studies’ heterogeneity to sampling error. Consequently, I^2^ expressed as the percentage of variability in the effect sizes not caused by sampling error was directly based on Cochran’s *Q*. Heterogeneity was graded as follows: no heterogeneity, I^2^ ≤ 25%; low heterogeneity, 25% < I^2^ ≤ 50%; moderate heterogeneity, 50% < I^2^ ≤ 75%; and high heterogeneity, I^2^ > 75% [30]. When I^2^ was > 50%, a restricted maximum likelihood random effect was applied; otherwise, a fixed effect was used.

Since GLN and antioxidant parameters were measured in different tissues (breast muscle, thigh muscle, and serum), a subgroup analysis was conducted to elucidate differences among tissues or to explain potential heterogeneity. In the case of I^2^ > 75%, a subgroup analysis was not possible (BWG, FI, and FCR) so a Baujat plot and influential case diagnostics were performed to detect outlying studies, and the pooled effect sizes were recalculated [31].

Meta-regressions with the glutamine concentration or the duration of the HS challenge as covariates were conducted to evaluate how they affected the response variables. The formula for the meta-regression was the following [29]:

(4) $${\hat{\theta }}_{K}=\theta +\beta x_{K}+\epsilon_{K}+\zeta_{K}$$

where:

θ is the overall true effect size;

${\hat{\theta }}_{K}$ is the observed effect size of the study *K;*

$x_{K}$ is the predictor or covariate with its regression coefficient β;

$\epsilon_{K}$ is the sampling error through which the effect size of a study deviates from its true effect; and

$\zeta_{K}$ denotes that even the true effect size of the study is only sampled from an overarching distribution of effect sizes. This error can also be referred to as the between-study heterogeneity.

Finally, publication bias was assessed by testing funnel plot asymmetry via Egger’s regression test.

## 3. Results

### 3.1. Study Selection Workflow

**Figure 2:**
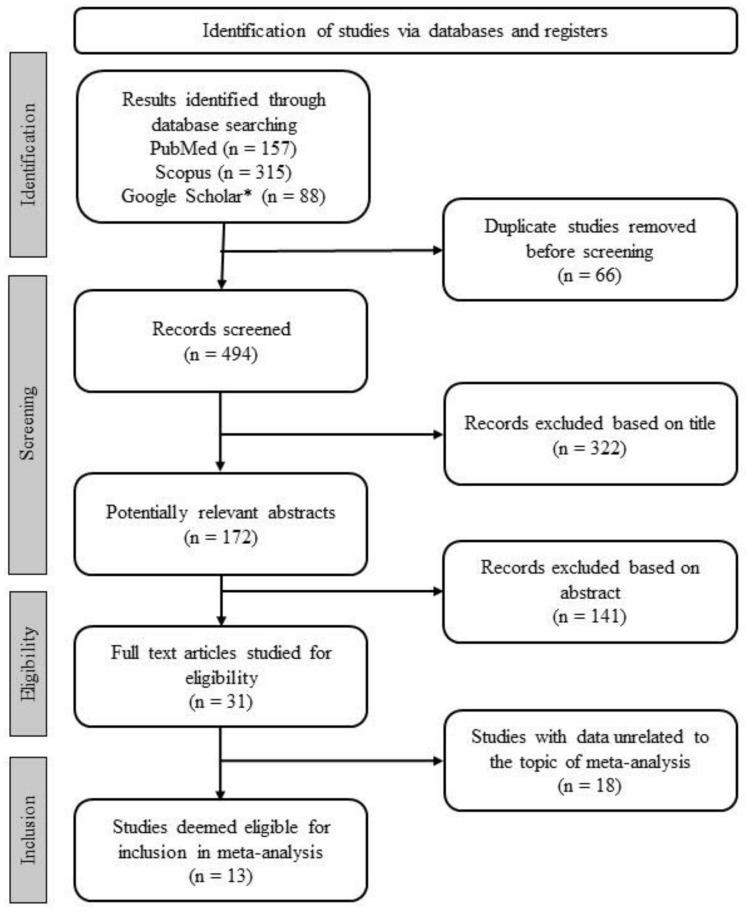
Flowchart of the study selection process. * The advanced function of Google Scholar was used to retrieve studies.

Figure 2 shows the flowchart of the study selection process. The database search resulted in 560 potentially eligible references. A total of 66 duplicates were removed, and then screenings were executed according to the inclusion criteria.

The title and abstract screening led to the exclusion of 322 and 141 records, respectively. Thus, 31 studies were found suitable for full-text reading. Finally, after the application of the inclusion criteria to the full texts, 18 studies were disqualified and a total of 13 studies were included in this systematic review. The exhaustive list of studies included in this meta-analysis is presented in Table 1.

### 3.2. Study Characteristics

The data were extracted from a total of 13 studies published from 2009 to 2020. Two breeds were used across all studies with a frequency as followed: Arbor acres (*n* = 11) and Ross 308 (*n* = 2). Studies evaluated three main categories of HS, namely chronic HS (*n* = 3), cyclic HS (*n* = 8), and acute HS (*n* = 2). The average peak temperature was about 33.5 °C, with a minimum of 28 °C and a maximum of 36 °C. Two studies considered applying the HS from one day old, while the remaining ones exposed the broilers from at least three weeks of age. Five different daily exposure periods were considered in the included studies: four studies applied HS for 24 h, one study applied HS for 12 h, two used 10 h of daily exposure, three maintained HS for 9 h, and finally, three other studies performed HS for 8 h.

**Figure 3:**
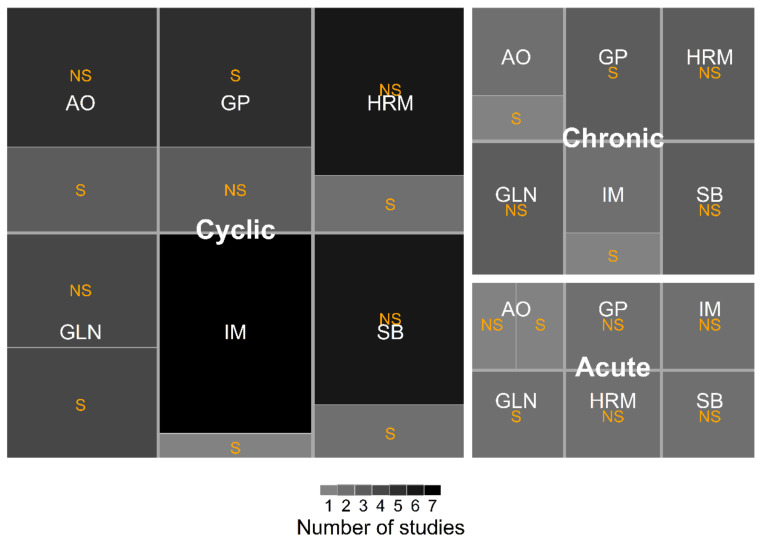
Treemap of outcomes evaluated from selected studies in the meta-analysis. The labels “Cyclic”, “Chronic” and “Acute”, are the different categories of HS applied in a study. The two or three-letter abbreviations correspond to a category of outcomes. The shade of gray refers to the number of reports that did or did not study a particular outcome in their trials. Abbreviations: AO: antioxidant parameters; GLN: dietary glutamine concentration; GP: growth performances; HRM: hormones; IM: intestinal morphology; NS: not studied; S: studied; SB: serum biochemicals.

Figure 3 displays the categories of outcomes studied across studies. Growth performances were by far the most reported (*n* = 8), and exclusively reported in studies evaluating cyclic or acute HS.

Moreover, six studies evaluated glutamine concentration (acute HS *n* = 2, cyclic HS *n* = 4), and five studies assessed antioxidant parameters (acute HS *n* = 1, chronic HS *n* = 1, cyclic HS *n* = 3). Serum biochemicals (cyclic HS *n* = 2), intestinal morphology (chronic HS *n* = 2), and hormones (cyclic HS *n* = 2) were equally reported.

### 3.3. Study Quality

Table 2 presents the risk of bias classification for the studies included in the meta-analysis.

No studies included in the review met the low risk of bias criteria from domains 1 to 10. Indeed, all studies presented an unclear risk of bias in domains 3, 4, 5, 7, and 8. One study was judged to have a high risk of bias relating to domain 6. Similarly, four other studies showed an unclear risk of bias regarding random outcome assessment. Fortunately, all the studies were deemed to present a low risk of bias concerning domains 1, 2, 9, and 10.

### 3.4. Effect of Dietary Glutamine on the Growth Performances of Broilers Reared under HS

As shown in Supplementary Figure S1, dietary supplementation of glutamine under HS increased the BWG of birds (SMD = 0.70, CI = 0.50 to 0.90, *p* < 0.05). Moderate heterogeneity was present in this effect since I^2^ = 67.3%, *p* < 0.001. Similarly, the diamond position in Supplementary Figure S2 reflects the overall positive effects of dietary glutamine supplementation on the FI in heat-stressed broilers. The effect was estimated at SMD = 0.64, CI = 0.43 to 0.86, and *p* < 0.05 with moderate heterogeneity (I^2^ = 67.4%, *p* < 0.001). Supplementary Figure S3 also reveals that the FCR of heat-stressed birds was significantly reduced (MD = −0.05, CI = −0.07 to −0.02, *p* < 0.05) by glutamine supplementation. Significant moderate heterogeneity was present in this effect (I^2^ = 63.5%, *p* < 0.001).

### 3.5. Effect of Dietary Glutamine on the Glutamine Concentration in Different Tissues of Broilers Reared under HS

Supplementary Figure S4 highlights that dietary supplementation of glutamine increased the glutamine concentration in broiler chickens, with an overall effect of SMD = 1.21, CI = 1.00 to 1.43, *p* < 0.05 with low heterogeneity (I^2^ = 25.3%, *p* = 0.17). The glutamine concentration in the breast muscle (SMD = 1.28, CI = 0.95 to 1.61, *p* < 0.05 and I^2^ = 62.3%, *p* < 0.01), serum (SMD = 1.33, CI = 0.82 to 1.85, *p* < 0.05 and I^2^ = 0.0%, *p* = 0.46), and thigh muscle (SMD = 1.08, CI = 0.73 to 1.43, *p* < 0.05 and I^2^ = 0.0%, *p* = 0.93) were all significantly increased.

### 3.6. Effect of Dietary Glutamine on the Antioxidant Status of Broilers Reared under HS

As described in Supplementary Figure S5, glutamine supplementation under HS increased the GPX levels (SMD = 1.12, CI = 0.73 to 1.51, *p* < 0.05) with moderate heterogeneity (I^2^ = 65.1%, *p* < 0.001). Specifically, GPX levels were increased in the breast muscle (SMD = 1.32, CI = 0.95 to 1.68, *p* < 0.05 and I^2^ = 0.0%, *p* = 0.81), and thigh muscle (SMD = 1.53, CI = 1.06 to 1.99, *p* < 0.05 and I^2^ = 30.4%, *p* = 0.24). In contrast, dietary glutamine did not increase GPX levels in the serum of heat-stressed broilers (SMD = 0.45, CI = −0.63 to 1.52, *p* = 0.41, and I^2^ = 83.4%, *p* = 0.24). The overall effects of dietary supplementation of glutamine on the SOD levels in broilers exposed to HS are depicted in Supplementary Figure S6. SOD levels were significantly increased in all tissues (SMD = 0.97, CI = 0.58 to 1.36, *p* < 0.05) with moderate heterogeneity (I^2^ = 60.9%, *p* < 0.001). A similar pattern was found concerning CAT (Supplementary Figure S7). The overall CAT levels were increased in all tissues (SMD = 0.94, CI = 0.72 to 1.16, *p* < 0.05) with low heterogeneity (I^2^ = 45.7%, *p* < 0.05). Furthermore, dietary supplementation of glutamine in heat-stressed birds (Supplementary Figure S8) resulted in an overall increase (SMD = 1.25, CI = 0.88 to 1.62, *p* < 0.05) in the GSH levels with moderate heterogeneity (I^2^ = 58.6%, *p* < 0.001).

Dietary glutamine led to significantly decreased MDA levels (SMD = −0.84, CI = −1.33 to −0.35, *p* < 0.01), which were especially strong in the serum (SMD = −0.83, CI = −1.25 to −0.41, *p* < 0.001), and thigh muscle (SMD = −1.30, CI = −1.86 to −0.74, *p* < 0.001). In contrast, MDA was not affected in the breast muscle (SMD = −0.38, CI = −1.64 to 0.89, *p* = 0.56) (Supplementary Figure S9). Although high heterogeneity was detected overall (I^2^ = 78.6%, *p* < 0.05), after subgroup analysis, it appears that the breast muscle subgroup contributed the most to the heterogeneity (I^2^ = 91.0%, *p* < 0.001) compared with the serum (I^2^ = 3.9%, *p* = 0.27) and thigh muscle (I^2^ = 53.1%, *p* = 0.06).

### 3.7. Effects of the Glutamine Concentration and HS Duration on Growth and Antioxidant Parameters

The models describing the effect of the HS duration on the response variables are presented in Table 3.

The HS duration effect was found to be significant for the FI, GPX, SOD, and CAT. Longer HS exposure resulted in decreased SMD of the FI (*p* < 0.01), GPX (*p* < 0.05), and SOD (*p* < 0.05), while increasing the CAT values (*p* < 0.05). The results of the glutamine concentration on the response variables are displayed in Table 4.

Significant results were found for the GPX and SOD levels. An increase in the glutamine concentration by 1% resulted in a rise in the GPX (*p* < 0.05). Similarly, SOD was increased (*p* < 0.05) when the dietary glutamine was increased by 1%.

### 3.8. Analysis of Bias

**Figure 4:**
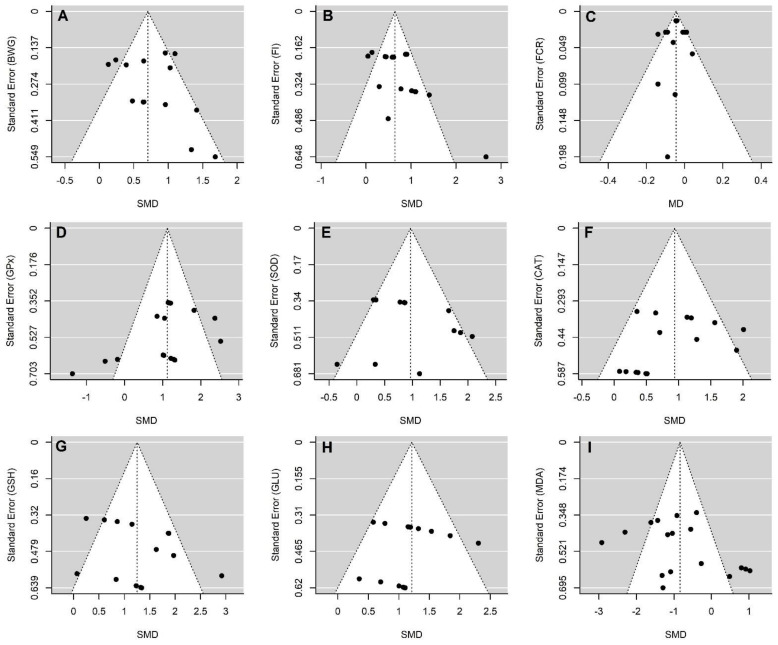
Standard error of funnel plots for the body weight gain (**A**), feed intake (**B**), feed conversion ratio (**C**), glutathione peroxidase (**D**), superoxide dismutase (**E**), catalase (**F**), glutathione (**G**), glutamine (**H**) and malondialdehydes (**I**). Abbreviations: BWG: body weight gain; CAT: catalase; FCR: feed conversion ratio; FI: feed intake; GLN: glutamine concentration in tissues; GPX: glutathione peroxidase; GSH: glutathione; MD: mean difference; MDA: malondialdehydes; SOD: superoxide dismutase; SMD: standardized mean difference. No evidence of publication bias was found for BWG (*p* = 0.377), FI (*p* = 0.106), FCR (*p* = 0.791), GLN (*p* = 0.662), GSH (*p* = 0.211), GPX (*p* = 0.072), SOD (*p* = 0.521), CAT (*p* = 0.287), and MDA (*p* = 0.226).

The publication bias was evaluated by assessing the asymmetry of funnel plots (Figure 4). Egger’s regression test parameters were calculated to assess funnel plot asymmetry. No evidence of publication bias was found for BWG (*p* = 0.377), FI (*p* = 0.106), FCR (*p* = 0.791), GLN (*p* = 0.662), GSH (*p* = 0.211), GPX (*p* = 0.072), SOD (*p* = 0.521), CAT (*p* = 0.287), and MDA (*p* = 0.226).

## 4. Discussion

**Figure 5:**
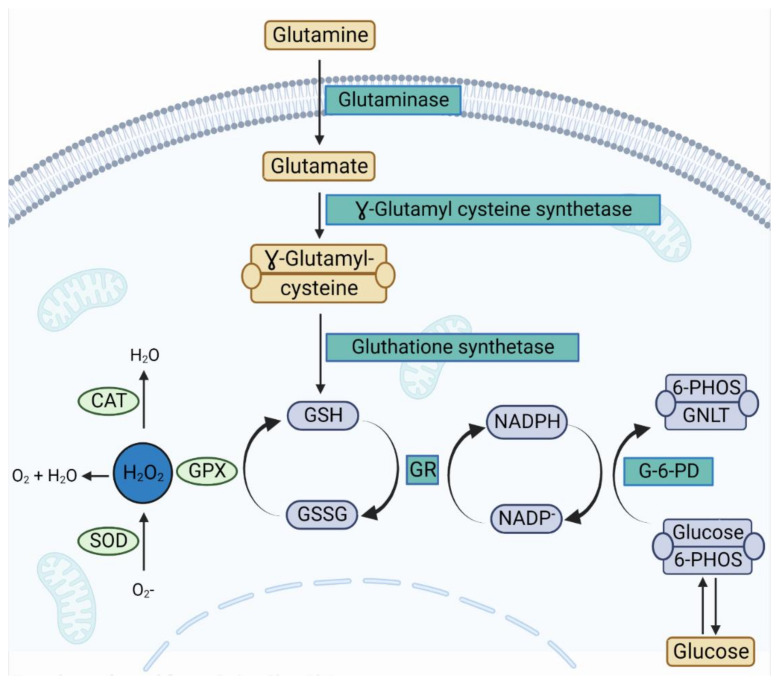
Involvement of glutamine in the antioxidant enzyme pathway. Glutamate is produced from glutamine via the action of phosphate-activated glutaminase. Thereafter, glutamate is converted into γ-glutamyl cysteine, which is the precursor of cellular glutathione (GSH). The three main antioxidant enzymes in the avian cell are glutathione peroxidase (GPX), superoxide dismutase (SOD), and catalase (CAT) [44]. GPX and CAT act synergistically to break down H_2_O_2_ into water after the preceding action of SOD, which converts O_2_^-^ into H_2_O_2_. The action of GPX is linked to several other enzymes such as glutathione reductase (GR), and glucose-6-phosphate dehydrogenase (G-6-PD). However, it also requires cofactors, namely GSH, NADPH, and glucose-6-phosphate for optimal efficiency. Cellular levels of glutathione disulfide (GSSG) increase when the action of GR is impaired, whereas the activity of GPX can be impaired following the lack of co-substrate GSH, leading to H_2_O_2_ toxicity [45].

Glutamine is an important amino acid involved in the regulation of protein synthesis and skeletal muscle growth [37]. The current findings suggest that the supplementation of glutamine enhances glutamine metabolism under HS. GLN is higher in all tissues, and overall SMD is 1.21, which is higher than 0.8 and can be considered a large effect [40]. Stressful conditions, such as injury and illness, are strongly correlated with depreciation in skeletal intracellular GLN [41]. In addition, lower GLN in tissues often leads to oxidative stress [42]. Thus, dietary glutamine can increase intracellular glutamine metabolism under HS since it is involved in redox signaling (Figure 5). More precisely, glutamine in the form of glutamate is a component of glutathione (GSH), which plays a key role in the redox state of myocytes [42,43].

Antioxidant enzymes and oxidative stress markers were among the most reported response variable categories in the studies included in the meta-analysis [16,23,32,33,38]. Supplementation of glutamine in heat-stressed birds led to significantly higher SMD in parameters such as SOD, CAT, and GSH, regardless of tissue, whereas GPX levels did not follow the same trend in serum. In fact, the subgroup analysis revealed that pooled SMD in serum GPX was associated with high heterogeneity. Statistical heterogeneity in a meta-analysis is strongly affected by the spread and precision of effect size estimates [29]. Data from two studies [32,33] were pooled to obtain an estimated SMD in serum. Interestingly, glutamine supplementation did not consistently influence the results of the same studies. While bird age, dietary glutamine concentrations, and the methodology used to assess GPX activity were relatively similar, one striking difference in study design was the duration of HS exposure (24 h/day for 42 days vs. 8 h/day for 14 days). Therefore, no perceptible impact of glutamine was detected when birds were exposed to 42 days [32], in contrast to 14 days [33] of HS. Likewise, meta-regression models highlighted a strong negative correlation between HS duration and GPX activity. It was also suggested that an exaggerated period of stress can compromise the restoration of oxidoreductive balance caused by permanent alterations in GPX activity [46]. Thus, some restorative abilities in birds regarding antioxidant defenses may be gradually lost following prolonged periods of HS. HS is well known to induce oxidative stress due to its impact on mitochondrial functions [47]. Oxidative stress is the state in which the organism can no longer neutralize the production of reactive oxygen species [48]. In poultry, the first line of antioxidant defense is constituted of the three major enzymes, namely, SOD, CAT, and GPX [44]. As previously explained, the antioxidant action of glutamine is reflected in its participation in the synthesis of GSH. As a result, GSH donates an electron to facilitate the action of GPX [49]. GPXs are a family of isoenzymes with oxidoreduction as the main function [50]. They were named after the enzyme GPX-1, which converts H_2_O_2_ resulting from the previous reaction of SOD into water [51]. Thus, this might explain why meta-regression models revealed that GPX and SOD levels simultaneously increased with higher dietary glutamine concentration. Furthermore, glutamine could increase SOD, CAT, and GPX levels in cells by modulating the nuclear factor erythroid 2-related 2/Kelch-like ECH-associated protein 1 (NRF2-Keap1) pathway [38]. The NRF2-Keap1 is a transcription factor pathway that activates the gene expression of the antioxidant response element gene cluster, including the antioxidant enzymes.

MDA is one of the end products of lipid peroxidation, and higher levels of MDA are indicative of substantial cell damage induced by free radicals in cell membrane phospholipids [52]. Interestingly, MDA levels were reduced in the serum and thigh muscle but not in the breast muscle of heat-stressed broilers after supplementation of glutamine. One possible explanation for this difference muscle-wise may have to do with their chemical compositions. Compared with thigh muscles, breasts were reported to contain a lower fat content [53]. In addition, the overall fatty acid profile of the breast muscle from fast-growing broilers was found to have a significantly lower percentage of polyunsaturated fatty acids (PUFAs) [54]. It is well known that lipid peroxidation preferentially oxidizes PUFAs [55,56]. Hence, heat-induced oxidative stress may lead to low levels of MDA production in the breast muscle. As such, glutamine supplementation may not be effective if no damage or very low damage occurs.

Arguably, the findings that the oxidative functions of HS-exposed birds were significantly enhanced by dietary glutamine supplementation are not surprising. The totality of antioxidant parameters, recording pooled SMDs largely superior to 0.8, suggests that glutamine supplementation leads to large effect sizes [40]. One hypothesis may explain such drastic improvements. It could be that the antioxidant-enhancing abilities of glutamine are exceptional and can be perceived in broilers reared under standard conditions or exposed to various other stressors. A research group indeed previously reported higher activities of SOD and GPX concomitant with lower MDA levels in the serum of broilers following 42 days of glutamine supplementation [57]. Similarly, the activities of all three major antioxidant enzymes were increased in the gut of broilers infected with *Salmonella Enteritidis* when they were fed diets enriched in glutamine [58]. Therefore, using glutamine as a mitigation strategy against HS, which is associated with pronounced alterations in the antioxidant system, might explain the magnitude of the effect sizes obtained.

It is already known that SMD and MD are the most common effect sizes considered when conducting a meta-analysis using continuous variables [59]. Our observations for growth performances, particularly BWG and FI, revealed pooled SMDs of 0.70 and 0.64, respectively. These values, according to Cohen [40], can be classified as medium effects and describe the effectiveness of dietary supplementation of glutamine in mitigating the deleterious effects of HS in broilers. Chickens lack sweat glands and therefore have a poor ability to regulate their body temperature in high-temperature environments [19]. HS was shown to reduce glutamine retention but increase its excretion and mobilization from tissues [34]. In broilers, decreased ability of the intestine to digest and absorb nutrients due to HS was one of the major causes of low BWG [60]. Additional glutamine was associated with beneficial effects such as promoting growth and development of the digestive system [36]. Supplementation of glutamine in the diet also maintained intestinal lymphocyte functions and increased metabolic activity and proliferation of enterocytes [61]. Furthermore, glutamine appeared to be linked with an increase in the intestinal villus height and number, as well as the reduction of pathogenic bacteria invasion [58]. Hence, supplementation of glutamine improves digestive function and compensates for the glutamine reserve depletion that occurs during HS.

The results of this meta-regression indicate that the improvement in FI induced by glutamine in heat-stressed broilers was time-dependent as the effect of glutamine supplementation decreased with longer HS exposure. The reason for this phenomenon may be acclimatization. A study evaluating the effects of the duration of HS on broilers reported a drastic difference in FI in the first week after heat exposure, but with a less noticeable effect at the end of the second week [62]. Another factor emphasizing this finding could be the category of HS evaluated in the studies included in the meta-analysis. In fact, cyclic HS was chosen in 72.7% of studies evaluating long-term HS. During cyclic HS, high-temperature periods alternate with normal rearing temperatures, increasing the coping ability of broilers over time [63]. During the heat period, birds tend to decrease their FI but are able to compensate thereafter. Therefore, broilers may become less sensitive to HS over time, and the benefits of supplementing glutamine may not diminish under HS.

According to the SYRCLE tool, all included studies contained an unclear risk of bias in at least one domain indicating that the current research does not account for certain biases. In fact, all studies presented an unclear risk of bias in the “blinding of caregivers” and “blinding of outcome assessor”. A proper definition of the methodology used to assess outcomes as well as having the evaluation performed by trained scientists can attenuate the impacts of these domains of bias [25]. Other areas also displayed unclear risk of bias in several studies with “random housing” and “allocation concealment” being the most prevalent. Many researchers tend to give limited details in their materials and methods section. Therefore, it is quite troublesome to evaluate the impacts of unclear risk of bias in a study without being speculative. In animal trials, proper assessment of the methodology is crucial because it may be a factor in deciding whether or not to include a study in a systematic review.

To the best of our knowledge, this review is the first to quantify the dietary response of heat-stressed broilers to GLN through a meta-analysis in which 13 studies are selected via a systematic review of the published literature up until June 2021. Like other systematic reviews, however, the current study has some limitations. First, only articles written in English were included in the meta-analysis for two specific reasons: (i) English is now used almost exclusively in the scientific community and is often referred to as the language of science [64]; (ii) translatability of articles published in other languages by computerized methods may result in errors as none of the authors are native speakers. Although the linguistic selection criteria can be considered as a limitation, a meta-analysis using these criteria seems to have a low possibility of bias [65,66]. After excluding papers on linguistic reasons, it was found that only 1 out of 36 consecutive meta-analyses could produce different results [66]. Another limitation may be in the subgroup analysis. In a subgroup analysis, the main purpose is to elucidate whether subgroups or categories have distinct effect sizes and whether there are significant differences between each subgroup [29]. It is also important to clarify that the selection and definition of subgroups are based on the aim and scope of the meta-analysis. The current study focused on how glutamine supplementation under HS might affect different samples (serum, breast, and thigh muscle), but did not evaluate the potential effects of parameters such as the authors’ country and broilers’ breed. In addition, these factors were not taken into account due to the disproportionate number of studies in some categories. For example, out of 13 studies, 11 were conducted in China and used the same breed.

## 5. Conclusions

This meta-analysis focused on the evaluation of the potential effects of dietary glutamine on heat-induced oxidative stress and growth performances in broilers. Regarding growth performances, dietary glutamine supplementation was found to increase FI and BWG while reducing FCR in heat-stressed broilers. Even though medium-to-high heterogeneity was present within the dataset, subgroup analysis was performed to detect the origin of the variation recorded in the effect sizes between studies. Some inconsistencies were detected in the antioxidant responses among tissues. While dietary glutamine enhanced antioxidant functions in most tissues, parameters such as GPX and MDA were unchanged in serum and breast muscle, respectively. Further analysis revealed that the duration of HS exposure was one of the main factors governing the effectiveness of glutamine supplementation. Outstanding results were noticed in trials exposing birds to short-term HS whereas the opposite was highlighted in trials with extended HS challenges. The systematic approach emphasized that HS is one of the most researched topics for the application of dietary glutamine. As depicted in this review, the antioxidant abilities of glutamine may help combat conditions such as infection, leaky gut, and exposure to other abiotic stressors. Although the studies included in the meta-analysis were performed with only two different broiler strains as subjects, similar outcomes might be expected as long as fast-growing strains are targeted. Another interesting perspective would be to conduct dose-response studies to detect potential interactions between glutamine supplementation levels and the duration of HS exposure whenever substantial data are available in the literature.

## Figures and Tables

**Table 1:** Characteristics of studies included in the meta-analysis.

Study (Authors and Year)	Breed	Sex	Country	HS Category	HS Starting Age (Days)	HS Ending Age (Days)	HS Duration in a Day (Hours)	Peak Temperature
Ayazi, 2014 [32]	Ross 308	-	Iran	Chronic	1	42	24	32 ± 1 °C
Bai et al., 2019 [33]	Arbor Acres	50% each	China	Cyclic	22	35	8	34 °C
Dai et al., 2011 [34]	Arbor Acres	Male	China	Cyclic	22	42	9	34 °C
Hu et al., 2015a [21]	Arbor Acres	Male	China	Cyclic	22	42	9	34 °C
Hu et al., 2015b [22]	Arbor Acres	Male	China	Cyclic	22	35	9	35 °C
Jazideh et al., 2014 [35]	Ross 308	Male	Iran	Chronic	1	42	24	32 ± 1 °C
Sifa et al., 2018 [16]	Arbor Acres	-	China	Acute	42	42	24	34 ± 1 °C
Wu et al., 2018 [9]	Arbor Acres	50% each	China	Cyclic	22	41	10	33 ± 1 °C
Wu et al., 2020 [36]	Arbor Acres	-	China	Cyclic	21	42	10	36 ± 1 °C
Dai et al., 2009 [37]	Arbor Acres	Male	China	Chronic	35	42	24	28 °C
Hu et al., 2020a [23]	Arbor Acres	-	China	Cyclic	22	42	8	34 °C
Hu et al., 2020b [38]	Arbor Acres	50% each	China	Cyclic	22	42	8	35 °C
Hu et al., 2016 [39]	Arbor Acres	Male	China	Acute	35	35	12	34 ± 1 °C

The HS categories can be defined as followed: acute: HS challenge of short duration (usually no more than 24 h); chronic: HS challenge of prolonged duration without variation in the temperature; cyclic: HS challenge of prolonged duration alternating period of high ambient and thermoneutral temperatures. Abbreviation: HS: heat stress.

**Table 2:** Risk of bias assessment according to the SYRCLE’s risk of bias tool for animal studies.

Studies	1	2	3	4	5	6	7	8	9	10
Ayazi, 2014 [32]	-	-	?	?	?	-	?	?	-	-
Bai et al., 2019 [33]	-	-	?	?	?	?	?	?	-	-
Dai et al., 2011 [34]	-	-	?	?	?	-	?	?	-	-
Hu et al., 2015a [21]	-	-	?	?	?	+	?	?	-	-
Hu et al., 2015b [22]	-	-	?	?	?	?	?	?	-	-
Jazideh et al., 2014 [35]	-	-	?	?	?	-	?	?	-	-
Sifa et al., 2018 [16]	-	-	?	?	?	-	?	?	-	-
Wu et al., 2018 [9]	-	-	?	?	?	-	?	?	-	-
Wu et al., 2020 [36]	-	-	?	?	?	-	?	?	-	-
Dai et al., 2009 [37]	-	-	?	?	?	-	?	?	-	-
Hu et al., 2020a [23]	-	-	?	?	?	?	?	?	-	-
Hu et al., 2020b [38]	-	-	?	?	?	?	?	?	-	-
Hu et al., 2016 [39]	-	-	?	?	?	-	?	?	-	-

Abbreviations: 1: sequence generation; 2: baseline characteristic; 3: allocation concealment; 4: random housing; 5: blinding (performance bias); 6: random outcome assessment; 7: blinding (detection bias); 8: incomplete outcome data; 9: selective outcome reporting; 10: other sources of bias; “-“: low risk; “?”: unclear risk; “+”: high risk.

**Table 3:** Meta-regression models for the influence of the heat stress duration on the response variables studied.

	BWG	FI	FCR	GLN	GPX	GSH	SOD	CAT	MDA
Intercept									
Estimate	1.039	1.077	−0.075	0.946	1.697	1.182	1.988	0.375	−0.397
SE	0.214	0.193	0.032	0.212	0.303	0.354	0.479	0.278	0.385
*p* value	<0.001	<0.001	0.021	<0.001	<0.001	0.001	<0.001	0.177	0.302
HS duration									
Estimate	−0.012	−0.017	0.001	0.024	−0.033	0.005	−0.045	0.004	−0.027
SE	0.007	0.006	0.001	0.015	0.014	0.022	0.019	0.017	0.018
*p* value	0.074	0.007	0.322	0.114	0.021	0.805	0.021	0.021	0.141
Model									
R^2^ (%)	9.48	40.4	4.47	14.5	24.71	0.09	43.09	33.83	9.88
τ^2^	0.098	0.074	0.001	0.065	0.33	0.334	0.157	0.11	0.76
I^2^ (%)	64.75	54.69	61.49	25.8	58.9	61.79	48.18	36.52	77.23

The significance of the effects of the heat stress duration is perceivable by the P-value associated with the HS duration for each variable evaluated. Similarly, the effect of longer heat stress duration on a variable is dependent on the sign of the HS duration estimate in the model of that specific variable: positive means increasing effect while negative means decreasing effect. Abbreviations: BWG: body weight gain; CAT: catalase; FCR: feed conversion ratio; FI: feed intake; GLN: glutamine concentration in tissues; GPX: glutathione peroxidase; GSH: glutathione; I^2^: percentage of variability in the effect sizes not caused by sampling error; MDA: malondialdehydes; R^2^: percentage of variation explained by the models; SE: standard error; SOD: superoxide dismutase; τ^2^: between-studies heterogeneity.

**Table 4:** Meta-regression models for the influence of the dietary glutamine concentration on the response variables studied.

	BWG	FI	FCR	GLN	GPX	GSH	SOD	CAT	MDA
Intercept									
Estimate	0.497	0.315	−0.524	1.424	0.41	0.662	0.194	0.428	−0.607
SE	0.261	0.221	0.034	0.271	0.402	0.421	0.422	0.333	0.557
*p* value	0.056	0.153	0.127	<0.001	0.308	0.115	0.645	0.198	0.275
GLNd									
Estimate	0.331	0.481	0.011	−0.244	0.703	0.54	0.828	0.462	−0.221
SE	0.384	0.296	0.049	0.269	0.348	0.352	0.414	0.282	0.478
*p* value	0.388	0.104	0.831	0.365	0.043	0.124	0.045	0.101	0.643
Model									
R^2^ (%)	0.05	18.33	0.07	0.57	24.72	16.08	36.88	40.96	0.08
τ^2^	0.112	0.102	0.001	0.076	0.33	0.247	0.174	0.098	0.89
I^2^ (%)	67.4	61.95	64.26	29.21	58.28	54.08	49.08	33.63	79.48

The significance of the effects of higher dietary glutamine concentration is perceivable by the P-value associated with the GLNd for each variable evaluated. Similarly, the effect of higher dietary glutamine concentration on a variable is dependent on the sign of the GLNd estimate in the model of that specific variable: positive means increasing effect while negative means decreasing effect. Abbreviations: BWG: body weight gain; CAT: catalase; FCR: feed conversion ratio; FI: feed intake; GLN: glutamine concentration in tissues; GLNd: dietary glutamine concentration; GPX: glutathione peroxidase; GSH: glutathione; I^2^: percentage of variability in the effect sizes not caused by sampling error; MDA: malondialdehydes; R^2^: percentage of variation explained by the models; SE: standard error; SOD: superoxide dismutase; τ^2^: between-studies heterogeneity.

## Data Availability

The data presented in this study are available on request from the corresponding author.

## Supplementary Materials

The following supporting information can be downloaded at: https://www.mdpi.com/article/10.3390/antiox12030570/s1, Figure S1: Forest plot of the standardized mean difference and 95% confidence interval of the effect of dietary glutamine supplementation on body weight gain in heat-stressed broilers. Figure S2: Forest plot of the standardized mean difference and 95% confidence interval of the effect of dietary glutamine supplementation on feed intake in heat-stressed broilers. Figure S3: Forest plot of the mean difference and 95% confidence interval of the effect of dietary glutamine supplementation on feed conversion ratio in heat-stressed broilers. Figure S4: Forest plot of the standardized mean difference and 95% confidence interval of the effect of dietary glutamine supplementation on glutamine tissue concentration in heat-stressed broilers. Figure S5: Forest plot of the standardized mean difference and 95% confidence interval of the effect of dietary glutamine supplementation on glutathione peroxidase tissue activity in heat-stressed broilers. Figure S6: Forest plot of the standardized mean difference and 95% confidence interval of the effect of dietary glutamine supplementation on superoxide dismutase tissue activity in heat-stressed broilers. Figure S7: Forest plot of the standardized mean difference and 95% confidence interval of the effect of dietary glutamine supplementation on catalase tissue activity in heat-stressed broilers. Figure S8: Forest plot of the standardized mean difference and 95% confidence interval of the effect of dietary glutamine supplementation on glutathione tissue concentration in heat-stressed broilers. Figure S9: Forest plot of the standardized mean difference and 95% confidence interval of the effect of dietary glutamine supplementation on malondialdehydes tissue concentration in heat-stressed broilers.
