# Supplementary material for: Effects of Dietary Glutamine Supplementation on Heat-Induced Oxidative Stress in Broiler Chickens: A Systematic Review and Meta-Analysis
Source: Antioxidants (Basel). 2023 Feb 24;12(3):570. doi: 10.3390/antiox12030570 (PMC10045030; doi:10.3390/antiox12030570)
Supplement: Supplementary file 1 [file antioxidants-12-00570-s001.zip › antioxidants-2148247-supplementary.pdf]

antioxidants

*Systematic Review*

# Effects of Dietary Glutamine Supplementation on Heat-Induced Oxidative Stress in Broiler Chickens: A Systematic Review and Meta-Analysis

**Chris Major Ncho[1,2,†], Vaishali Gupta[1,2,†] and Yang-Ho Choi[1,2,3*]**

[1] Department of Animal Science, Gyeongsang National University, Jinju 52828 and Republic of Korea
[2] Institute of Agriculture and Life Sciences, Gyeongsang National University, Jinju 52828, Republic of Korea
[3] Division of Applied Life Sciences (BK21 Plus Program), Gyeongsang National University, Jinju 52828, Republic of Korea
† These authors contributed equally to this work.
* Correspondence: yhchoi@gnu.ac.kr

**Citation:** Ncho, C.M.; Gupta, V.; Choi, Y.-H. Effects of Dietary Glutamine Supplementation on Heat-Induced Oxidative Stress in Broiler Chickens: A Systematic Review and Meta-Analysis. *Antioxidants* **2023**, *12*, 570. https://doi.org/10.3390/antiox12030570

Academic Editor: Stanley Omaye

Received: 23 December 2022
Revised: 22 February 2023
Accepted: 23 February 2023
Published: 24 February 2023

**Copyright:** © 2023 by the authors. Licensee MDPI, Basel, Switzerland. This article is an open access article distributed under the terms and conditions of the Creative Commons Attribution (CC BY) license (https://creativecommons.org/licenses/by/4.0/).

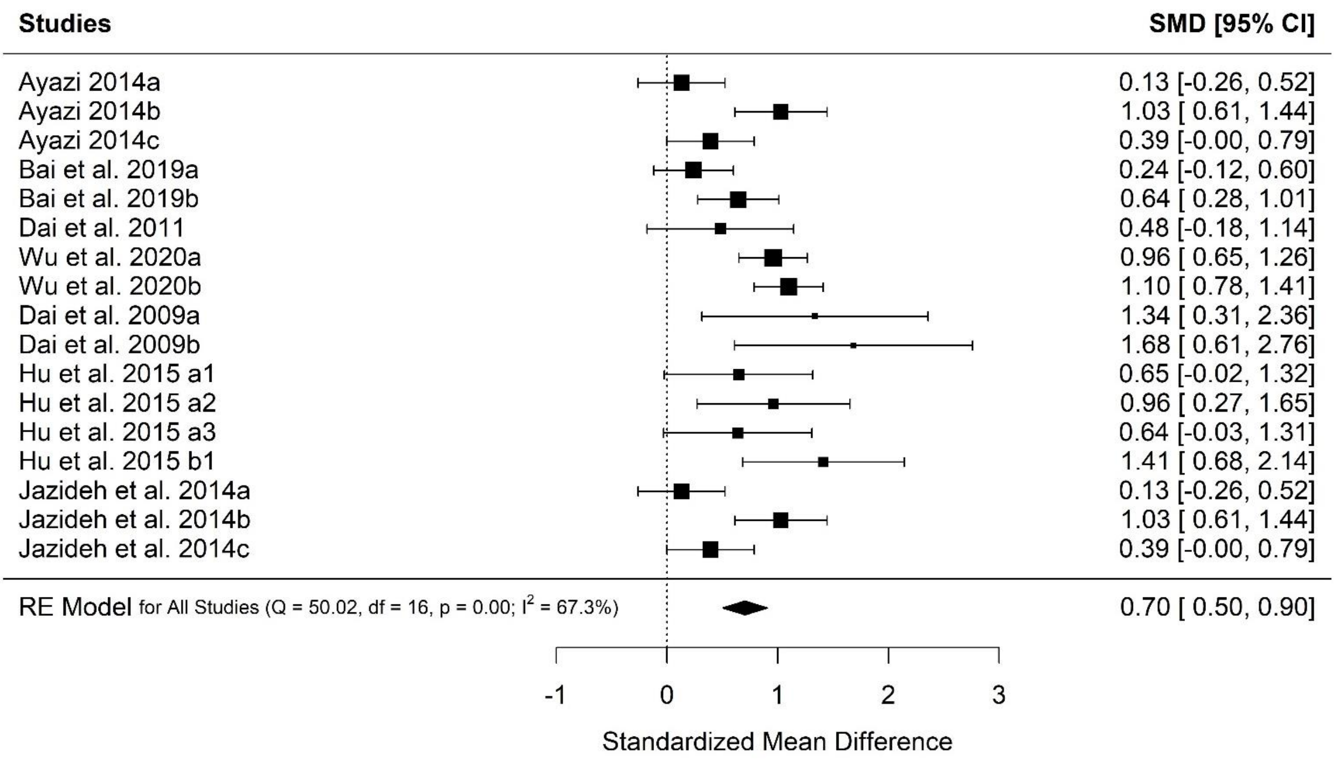


**Supplementary Figure S1.** Forest plot of the standardized mean difference and 95% confidence interval of the effect of dietary glutamine supplementation on body weight gain in heat-stressed broilers. Letters at the end of a study name (a,b..) indicate treatments included within the same study. When two studies were published by the same author during the same year and included more than one treatment, letters, and numbers (a1,a2,b1,b2,…) were added at the end of the studies. The black squares and their intervals refer respectively to the effect sizes and 95% confidence intervals associated with each study. The black diamond indicates the pooled effect size from all the studies. Abbreviations: CI: confidence interval, df: degrees of freedom, RE: random effect, SMD: standardized mean difference.

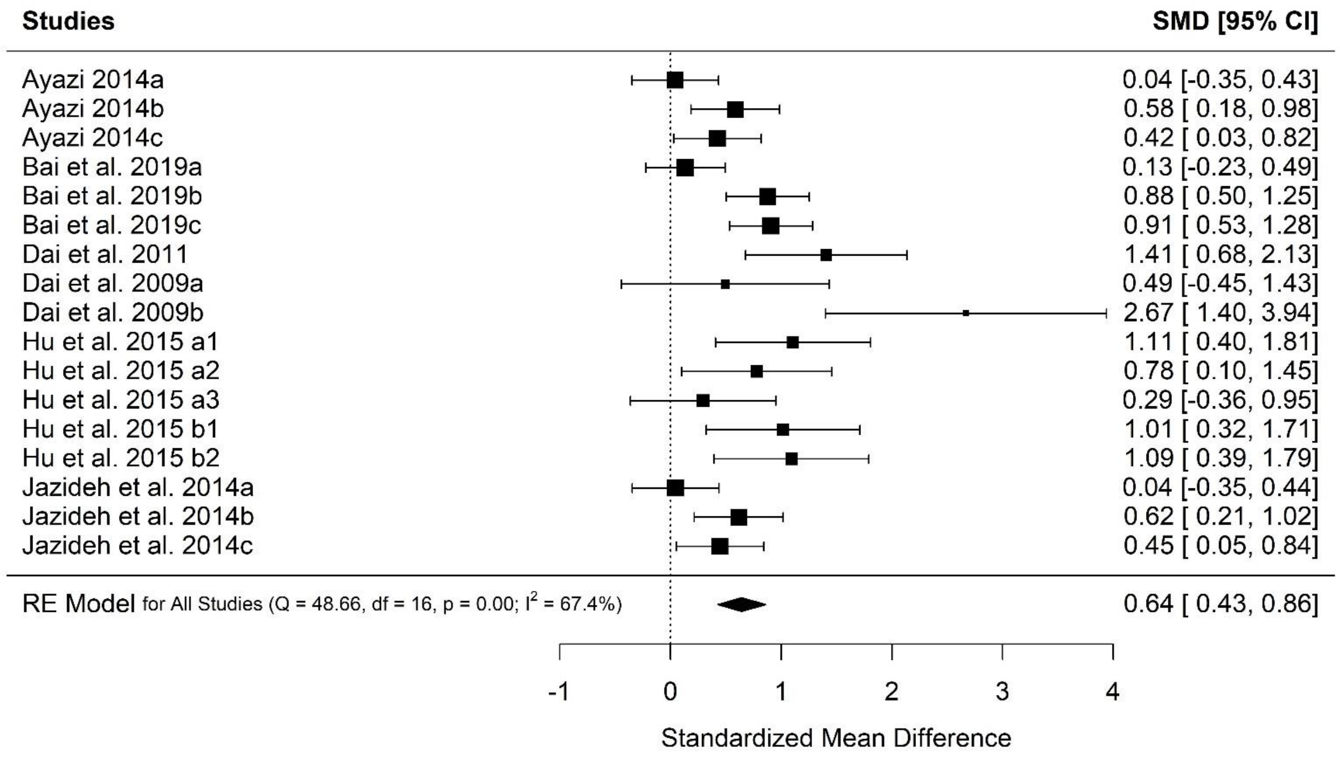


**Supplementary Figure S2.** Forest plot of the standardized mean difference and 95% confidence interval of the effect of dietary glutamine supplementation on feed intake in heat-stressed broilers. Letters at the end of a study name (a,b..) indicate treatments included within the same study. When two studies were published by the same author during the same year and included more than one treatment, letters, and numbers (a1,a2,b1,b2,…) were added at the end of the studies. The black squares and their intervals refer respectively to the effect sizes and 95% confidence intervals associated with each study. The black diamond indicates the pooled effect size from all the studies. Abbreviations: CI: confidence interval, df: degrees of freedom, RE: random effect, SMD: standardized mean difference.

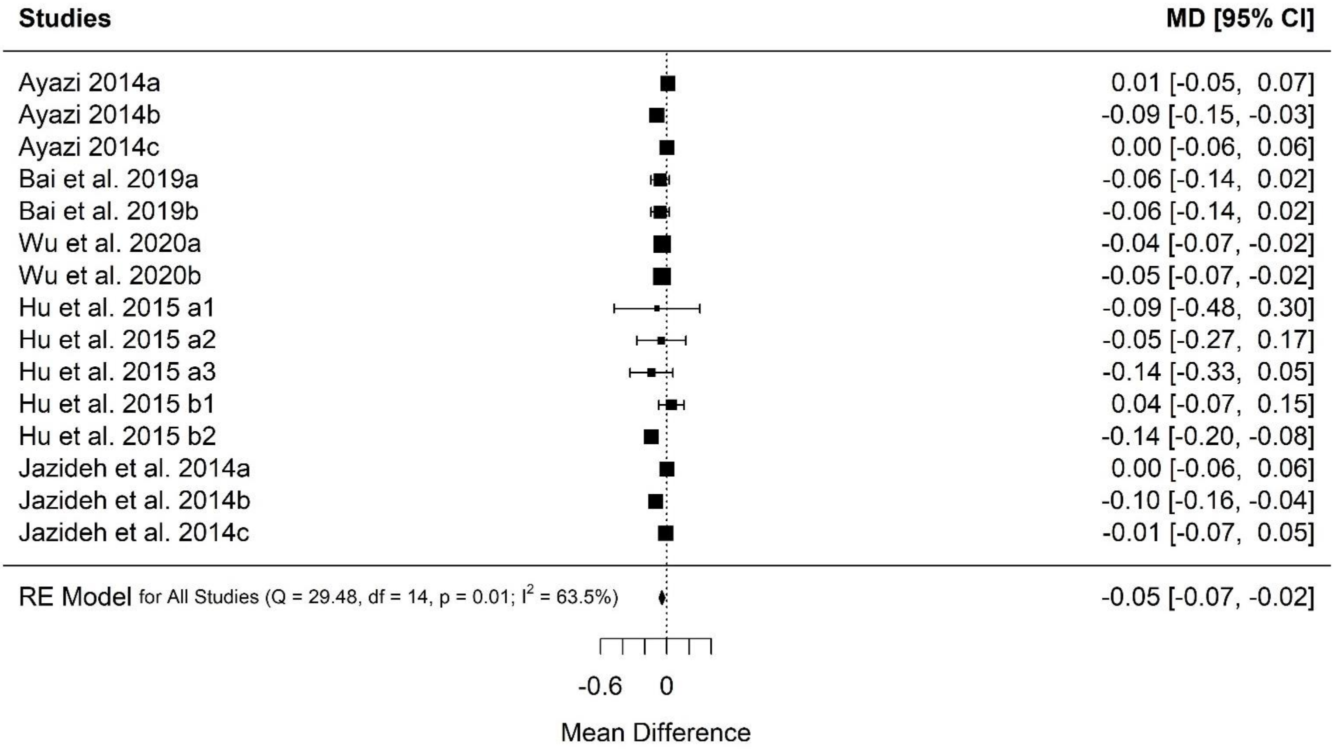


**Supplementary Figure S3.** Forest plot of the mean difference and 95% confidence interval of the effect of dietary glutamine supplementation on feed conversion ratio in heat-stressed broilers. Letters at the end of a study name (a,b..) indicate treatments included within the same study. When two studies were published by the same author during the same year and included more than one treatment, letters, and numbers (a1,a2,b1,b2,…) were added at the end of the studies. The black squares and their intervals refer respectively to the effect sizes and 95% confidence intervals associated with each study. The black diamond indicates the pooled effect size from all the studies. Abbreviations: CI: confidence interval, df: degrees of freedom, FE: fixed effect, MD: mean difference.

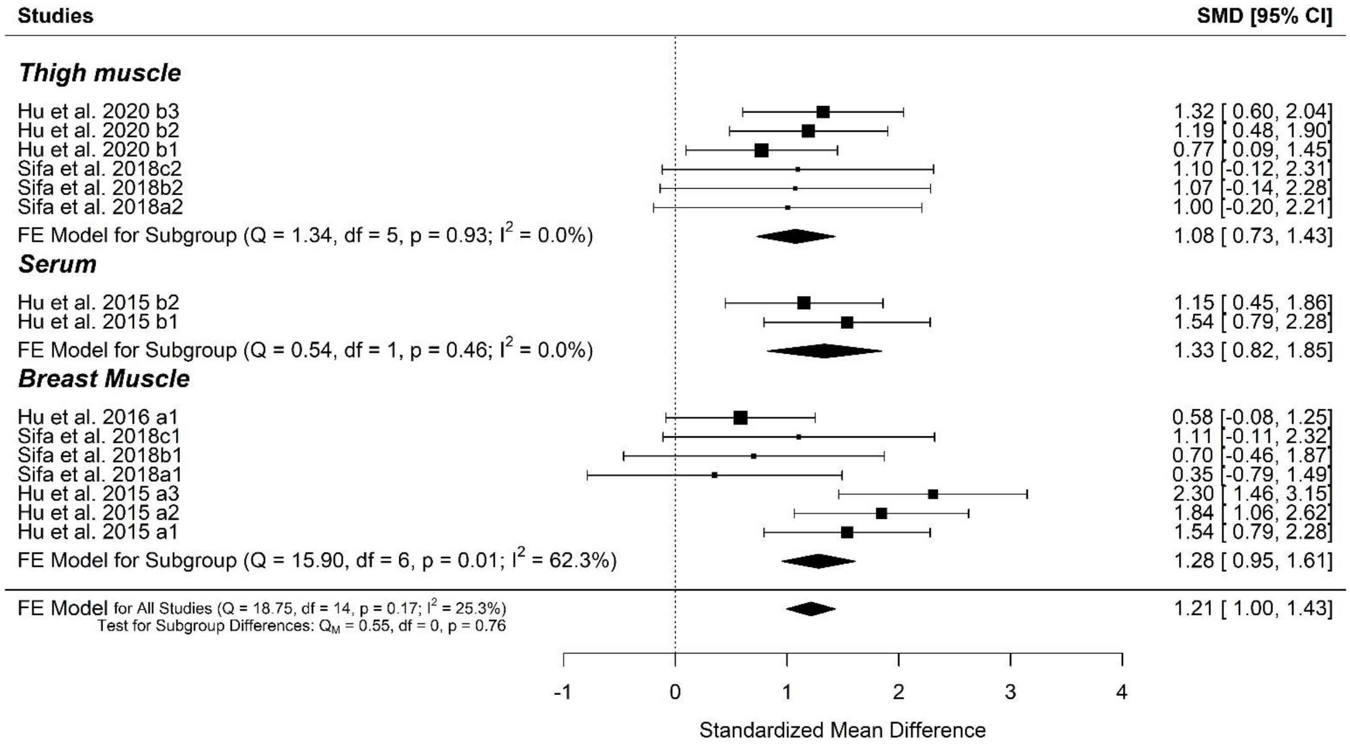


**Supplementary Figure S4.** Forest plot of the standardized mean difference and 95% confidence interval of the effect of dietary glutamine supplementation on glutamine tissue concentration in heat-stressed broilers. Letters at the end of a study name (a,b..) indicate treatments included within the same study. When two studies were published by the same author during the same year and included more than one treatment, letters, and numbers (a1,a2,b1,b2,…) were added at the end of the studies. The black squares and their intervals refer respectively to the effect sizes and 95% confidence intervals associated with each study. The black diamond indicates the pooled effect size from all the studies. Abbreviations: CI: confidence interval, df: degrees of freedom, FE: fixed effect, SMD: standardized mean difference.

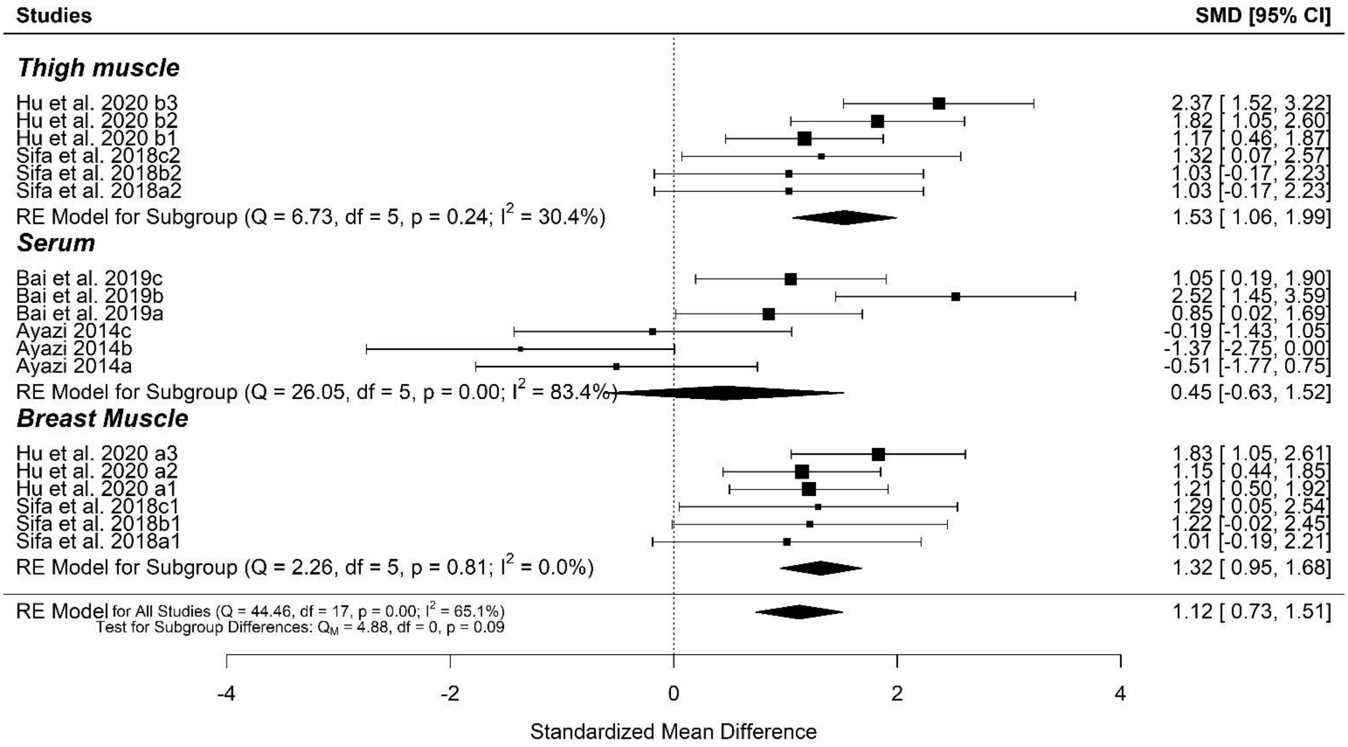


**Supplementary Figure S5.** Forest plot of the standardized mean difference and 95% confidence interval of the effect of dietary glutamine supplementation on glutathione peroxidase tissue activity in heat-stressed broilers. Letters at the end of a study name (a,b..) indicate treatments included within the same study. When two studies were published by the same author during the same year and included more than one treatment, letters, and numbers (a1,a2,b1,b2,…) were added at the end of the studies. The black squares and their intervals refer respectively to the effect sizes and 95% confidence intervals associated with each study. The black diamond indicates the pooled effect size from all the studies. Abbreviations: CI: confidence interval, df: degrees of freedom, RE: random effect, SMD: standardized mean difference.

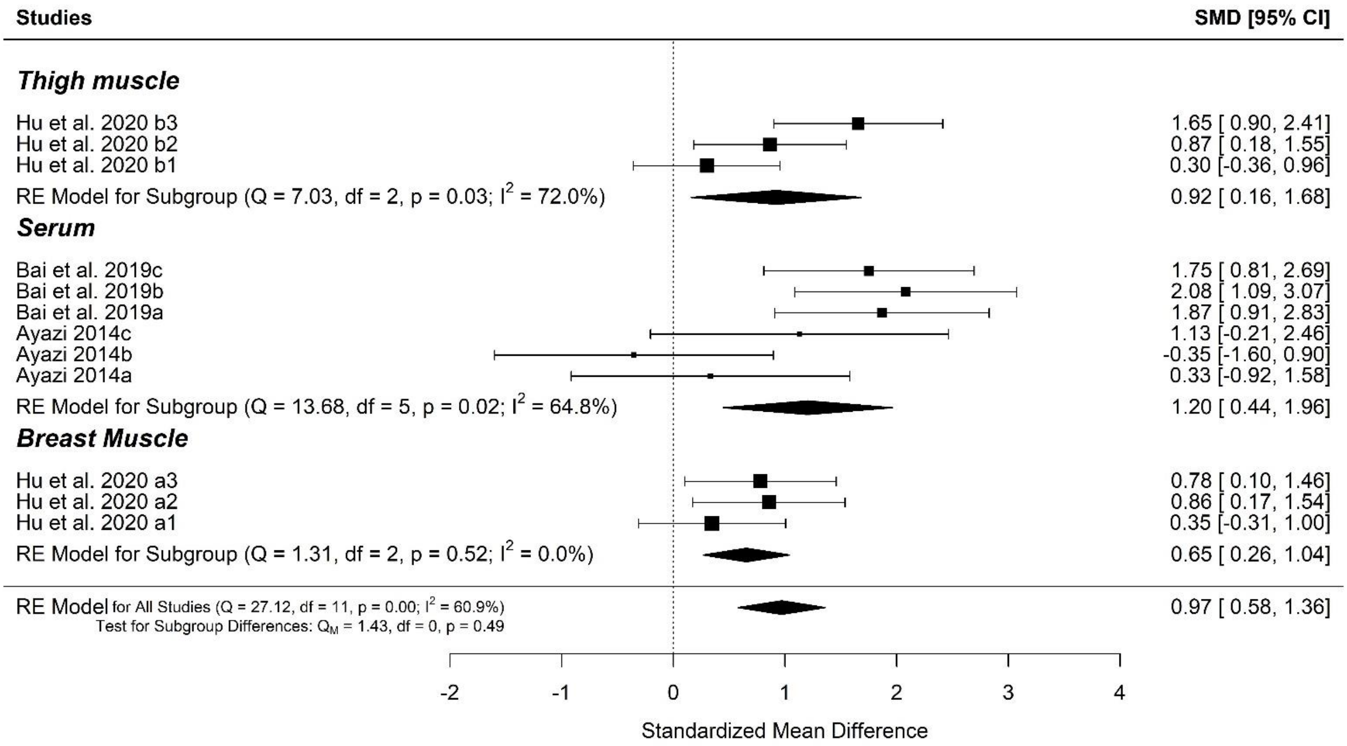


**Supplementary Figure S6.** Forest plot of the standardized mean difference and 95% confidence interval of the effect of dietary glutamine supplementation on superoxide dismutase tissue activity in heat-stressed broilers. Letters at the end of a study name (a,b..) indicate treatments included within the same study. When two studies were published by the same author during the same year and included more than one treatment, letters, and numbers (a1,a2,b1,b2,…) were added at the end of the studies. The black squares and their intervals refer respectively to the effect sizes and 95% confidence intervals associated with each study. The black diamond indicates the pooled effect size from all the studies. Abbreviations: CI: confidence interval, df: degrees of freedom, RE: random effect, SMD: standardized mean difference.

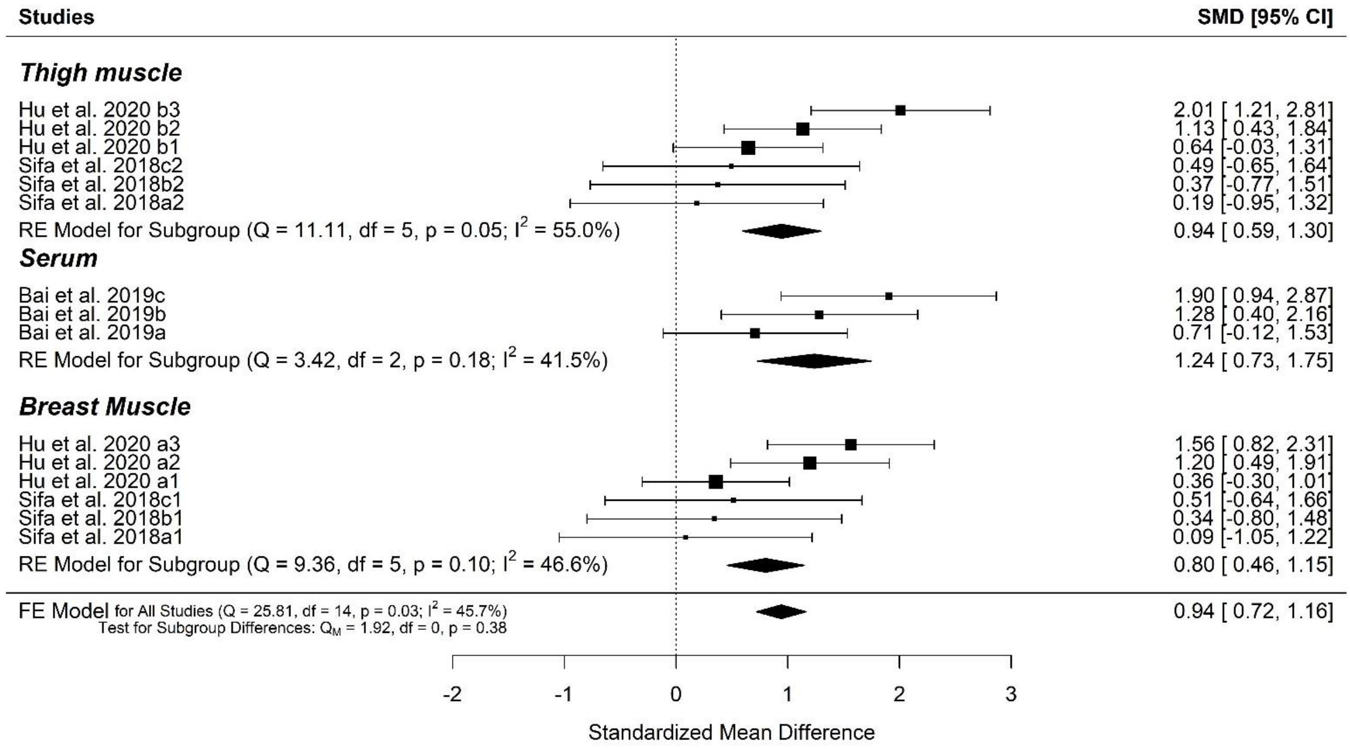


**Supplementary Figure S7.** Forest plot of the standardized mean difference and 95% confidence interval of the effect of dietary glutamine supplementation on catalase tissue activity in heat-stressed broilers. Letters at the end of a study name (a,b..) indicate treatments included within the same study. When two studies were published by the same author during the same year and included more than one treatment, letters, and numbers (a1,a2,b1,b2,…) were added at the end of the studies. The black squares and their intervals refer respectively to the effect sizes and 95% confidence intervals associated with each study. The black diamond indicates the pooled effect size from all the studies. Abbreviations: CI: confidence interval, df: degrees of freedom, FE: fixed effect, SMD: standardized mean difference.

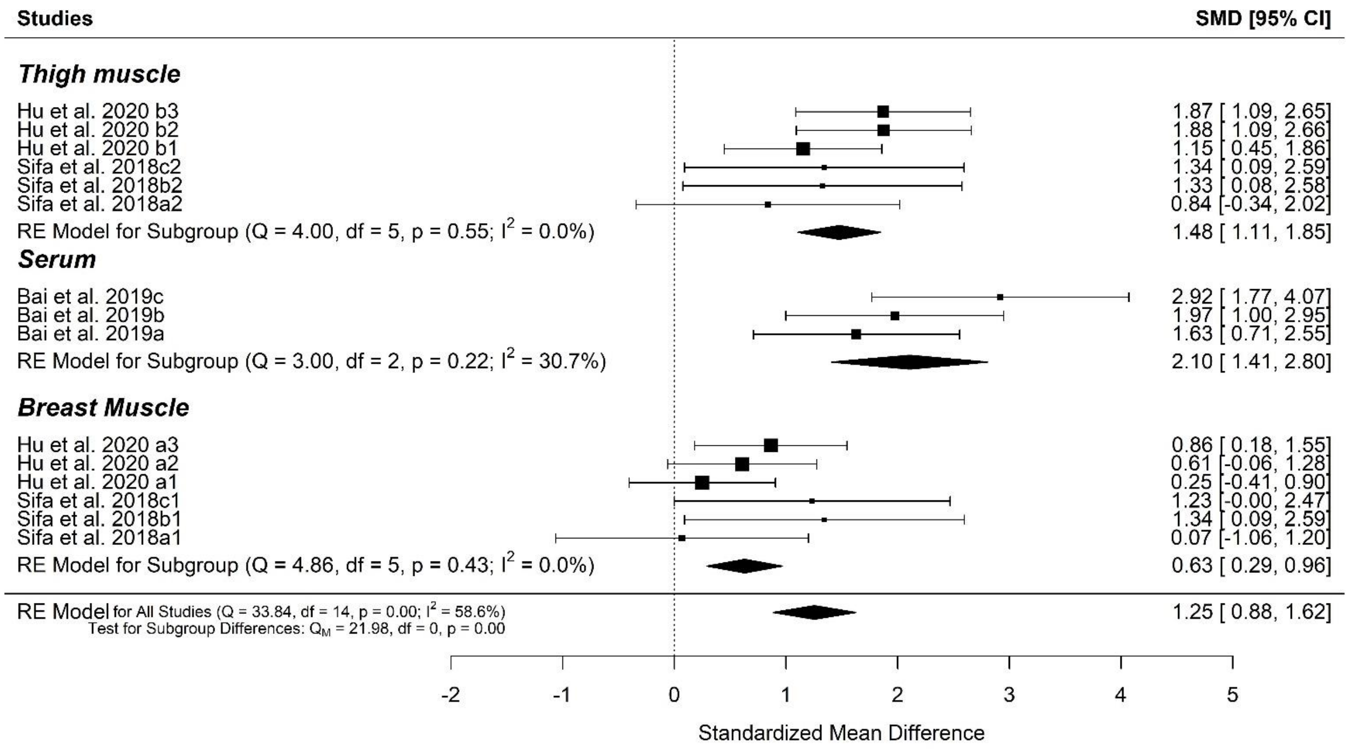


**Supplementary Figure S8.** Forest plot of the standardized mean difference and 95% confidence interval of the effect of dietary glutamine supplementation on glutathione tissue concentration in heat-stressed broilers. Letters at the end of a study name (a,b..) indicate treatments included within the same study. When two studies were published by the same author during the same year and included more than one treatment, letters, and numbers (a1,a2,b1,b2,…) were added at the end of the studies. The black squares and their intervals refer respectively to the effect sizes and 95% confidence intervals associated with each study. The black diamond indicates the pooled effect size from all the studies. Abbreviations: CI: confidence interval, df: degrees of freedom, RE: random effect, SMD: standardized mean difference.

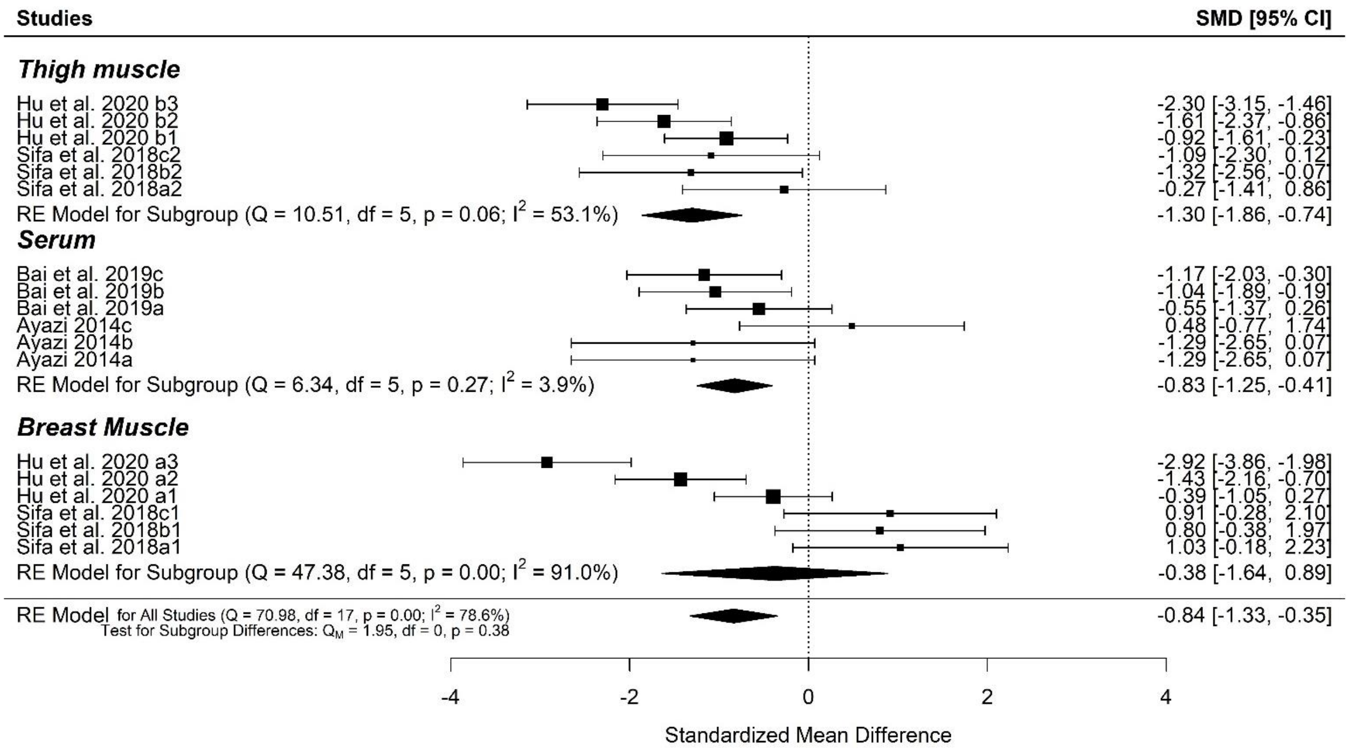


**Supplementary Figure S9.** Forest plot of the standardized mean difference and 95% confidence interval of the effect of dietary glutamine supplementation on malondialdehydes tissue concentration in heat-stressed broilers. Letters at the end of a study name (a,b..) indicate treatments included within the same study. When two studies were published by the same author during the same year and included more than one treatment, letters, and numbers (a1,a2,b1,b2,…) were added at the end of the studies. The black squares and their intervals refer respectively to the effect sizes and 95% confidence intervals associated with each study. The black diamond indicates the pooled effect size from all the studies. Abbreviations: CI: confidence interval, df: degrees of freedom, RE: random effect, SMD: standardized mean difference.
